# Steroidogenic activity of liposomal methylated resveratrol analog 3,4,5,4′-tetramethoxystilbene (DMU-212) in human luteinized granulosa cells in a primary three-dimensional in vitro model

**DOI:** 10.1007/s12020-023-03458-9

**Published:** 2023-08-12

**Authors:** Małgorzata Józkowiak, Dominik Kobylarek, Artur Bryja, Justyna Gogola-Mruk, Mikołaj Czajkowski, Paulina Skupin-Mrugalska, Bartosz Kempisty, Robert Z. Spaczyński, Hanna Piotrowska-Kempisty

**Affiliations:** 1 Department of Toxicology, Poznan University of Medical Sciences, Poznan, Poland; 2 Doctoral School, Poznan University of Medical Sciences, Poznan, Poland; 3 Department of Neurology, Poznan University of Medical Sciences, Poznan, Poland; 4 Division of Anatomy, Department of Human Morphology and Embryology, Wroclaw Medical University, Wroclaw, Poland; 5 Laboratory of Physiology and Toxicology of Reproduction, Institute of Zoology and Biomedical Research, Jagiellonian University, Krakow, Poland; 6 Department of Inorganic & Analytical Chemistry, Collegium Pharmaceuticum, Poznan University of Medical Sciences, Poznan, Poland; 7 Prestage Department of Poultry Sciences, North Carolina State University, Raleigh, NC 27695 USA; 8 Department of Veterinary Surgery, Institute of Veterinary Medicine, Nicolaus Copernicus University in Torun, Torun, Poland; 9 Center for Gynecology, Obstetrics and Infertility Treatment Pastelova, Poznan, Poland; 10 Department of Basic and Preclinical Sciences, Institute of Veterinary Medicine, Nicolaus Copernicus University in Torun, Torun, Poland

**Keywords:** Human ovarian granulosa cells, Resveratrol, DMU-212, Liposomes, Steroid hormone secretion

## Abstract

**Purpose:**

Steroid hormone secretion is one of the key functions of granulosa cells (GCs). Resveratrol is a natural polyphenol, known for its beneficial health effects, such as improving reproductive health. However, its application is limited due to poor bioavailability. The methoxy derivative of resveratrol (DMU-212) was demonstrated to be more lipophilic, and therefore of greater bioavailability. However, since the addition of methoxy groups to the stilbene scaffold was found to make the molecule insoluble in water, DMU-212 was loaded into liposomes. This study aimed to evaluate how the liposomal formulation of DMU-212 (lipDMU-212) alters estradiol and progesterone secretion of human ovarian GCs in a primary three-dimensional cell culture model.

**Methods:**

DMU-212-loaded liposomes were prepared by thin film hydration followed by extrusion. Cell viability was measured after exposure of GCs spheroids to the liposomal formulation of DMU-212 using CellTiter-Glo^®^ 3D Cell Viability Assay. The secretion of estradiol and progesterone was determined using commercial ELISA kits. RT-qPCR was conducted to analyze the expression of steroidogenesis-related genes. Finally, the western blot technique was used to analyze the effect of lipDMU-212 and FSH treatments on CYP11A1 and HSD3B1 protein levels.

**Results:**

lipDMU-212 was found to significantly increase estradiol and progesterone secretion in a dose-dependent manner by enhancing the expression of *CYP11A1, HSD3B1, StAR, CYP17A1, CYP19A1*, and *HSD17B1* genes. We have also shown that lipDMU-212, used alone and in combination with FSH, significantly increased the expression of the HSD3B1 and CYP11A1 proteins in GCs. Furthermore, our study suggests that lipDMU-212 increases FSH activity.

**Conclusions:**

This is the first study to describe the steroidogenic activity of liposomal formulation of DMU-212, possibly through increasing the *StAR* and *CYP19A1* expression. These findings suggest that lipDMU-212 might have a beneficial effect in the treatment of disorders related to estrogen deficiency and hyperandrogenism, such as PCOS.

## Introduction

GCs form an important component of the ovary since they play a key role in the production of steroid hormones, mainly estradiol. The synthesis of hormones requires close cooperation between GCs and theca cells, which surround the follicle and produce androgens following luteinizing hormone (LH) stimulation. Once androgens diffuse into GCs, aromatase (CYP19A1) converts them to estrogens in response to follicle-stimulating hormone (FSH) [1]. Any disruptions in these tightly regulated processes may lead to abnormal patterns of steroid production and consequently to polycystic ovary syndrome (PCOS), which is the most common disorder related to hyperandrogenism and arrested follicular development [2]. Resveratrol (3,4’,5-trans-trihydroxystilbene) is a natural polyphenol that is mainly present in grape skin. Due to its extraordinary properties, resveratrol is widely used in health improvement and longevity. Recently, the utilization of resveratrol in ovarian dysfunction therapy has been discussed.

Several studies have revealed that resveratrol improves ovarian functions, promotes the development of ovarian follicles, protects GCs from degeneration, and enhances their proliferation and differentiation [3–7]. However, despite the relatively high absorption of resveratrol, its rapid and extensive biotransformation as well as fast elimination from the body result in bioavailability of less than 1% [8].

According to the structure-biological activity studies, the substitution of the hydroxyl (-OH) groups with methoxy (-OMe) ones in resveratrol may enhance the lipophilicity of the compound, making it more bioavailable. On the other hand, the introduction of methoxy groups to the stilbene scaffold has simultaneously been found to influence the solubility of the molecule [9].

Hence, liposomes that can be loaded with compounds of different lipophilic-hydrophilic nature provide a feasible means to achieve the greater therapeutic potential of methoxy derivatives.

Among several methoxy derivatives of resveratrol, 3,4,5,4′-tetramethoxystilbene (DMU-212) has been reported to exert potent anticancer activity in several cancer cell lines [10–21]. Furthermore, the cytotoxic activity of DMU-212 has been revealed to be associated with the expression level of cytochrome P450 CYP1A1 enzyme, which is overexpressed in various types of human cancer compared to normal tissue [22, 23]. Piotrowska-Kempisty et al. have shown no cytotoxic effect of DMU-212 against ovarian noncancerous HOSE cells without CYP1A1, as compared to their ovarian cancerous counterparts with the expression of this protein [24]. Therefore, DMU-212 might be suggested not to exert any cytotoxicity in human noncancerous GCs.

So far, information regarding the role of resveratrol analogs in the regulation of ovarian steroidogenesis is limited. To the best of our knowledge, there is only one publication concerning the effect of hydroxylated and methylated resveratrol analogs on GCs, however, in a porcine in vitro model [25]. Since the administration of DMU-212-loaded liposomes may be of clinical relevance due to improved pharmacokinetics, this is of great importance to understand the mechanisms driving its steroidogenic activity in human GCs.

The study aims to evaluate how liposomal formulation of DMU-212 affects estradiol and progesterone secretion of human ovarian GCs in a primary three-dimensional (3D) cell culture model.

## Materials and methods

### Chemicals and reagents

All chemicals used were of analytical grade unless stated otherwise. DMU-212 was purchased from Sigma-Aldrich Co. (St. Louis, MO, USA). 1-palmitoyl-2-oleoyl-*sn*-glycero-3-phosphocholine (POPC), 1-palmitoyl-2-oleoyl-*sn*-glycero-3-phospho-(1’-rac-glycerol) (sodium salt) (POPG) were acquired from Avanti Polar Lipids (Alabaster, AL, USA). Chloroform and acetonitrile were purchased from Avantor Performance Materials Inc. (Radnor, PA, USA). The CellTiter-Glo® 3D Cell Viability Assay Kit was provided by Promega Co. (Madison, WI, USA). Estradiol and Progesterone ELISA kits were acquired from DRG Instruments GmbH (Marburg, Germany). Super Signal West Pico PLUS Chemiluminescent Substrate was purchased from Thermo Fisher Scientific (Waltham, Massachusetts, USA). The Trans-Blot Turbo Mini 0.2 µm PVDF Transfer Packs set and the Mini-PROTEAN® TGX™ Precast Gels were provided by Bio-Rad (Hercules, California, USA). All other materials were purchased from Sigma-Aldrich Co. (St. Louis, MO, USA) unless otherwise stated.

### Liposomes formulation and characteristics

Liposomes were prepared by thin film hydration (TLH) followed by extrusion. Chloroform solutions of POPC (50 mg/mL), POPG (50 mg/mL), and DMU-212 (10 mg/mL) were prepared. Volumes of stock solutions were mixed in a glass round-bottom flask. Chloroform was evaporated gradually under reduced pressure at 40 °C in a rotary evaporator (Heidolph GmbH, Schwalbach, Germany). The resulting lipid film was hydrated using mPBS (Dulbecco’s Phosphate Buffered Saline) in a rotary evaporator. The resulting liposome suspension was passed 21 times through polycarbonate membrane equipped with 10 mm drain discs (Whatman, Kent, UK) with pore diameters of 100 nm using a syringe extruder (Avanti Polar Lipids, Alabaster, AL, USA). Unbound material was separated from liposomes by ultrafiltration using Amicon Ultra centrifugal filters with molecular weight cut-off (MWCO) 50 kDa (Merck KGa, Darmstadt, Germany). Liposome samples were stored at 2–8 °C, protected from light.

The size and zeta potential of liposomes were determined by dynamic light scattering (DLS) laser Doppler electrophoresis using Malvern Zetasizer Nano ZS (Malvern Instruments Ltd., Malvern, UK), as described elsewhere [26].

Concentration of DMU-212 in liposomal formulation was determined by High-Performance Liquid Chromatography (HPLC) at 40 °C. Liposome samples were diluted 5 times in methanol prior to analysis. The analysis was carried out using Agilent 1260 Infinity II(Agilent Technologies, Santa Clara, CA, USA) equipped with a DAD detector. The chromatographic separation was achieved on Luna Omega PS C18 column (150 mm × 4.6 mm I.D., 3 µm particle size) (Phenomenex Inc, Torrance, CA, USA). A mobile phase gradient of eluent A (water with 5 mM ammonium acetate and 0.1% formic acid) and eluent B (acetonitrile) was applied at 0.8 mL/min. The optimized gradient profile was the following: 60% A was maintained for 3 min, declined to 10% A in 4 min, kept at 10% A for 2 min, then reversed to the original composition of 60% A over 1 min and remained constant for 5 min to re-equilibrate the column [27]. Detection was carried out at 325 nm and DMU-212 was eluted at 9.7 min. Encapsulation efficiency, EE (%), was calculated according to Eq. (1): EE = (Cm/Ci) × 100%, where Cm is concentration of DMU-212 loaded into liposomes, determined by HPLC, Ci is the maximum concentration of DMU-212 assumed at 100% EE. OpenLAB CDS ChemStation Edition Rev. C.01.07 SR3 was used for data acquisition and processing.

### Source and culture of human ovarian granulosa cells

Human luteinized GCs were isolated from follicular fluid obtained from 20 women, aged 25-40 years old, undergoing IVF-ICSI procedure, due to tubal or male factor infertility, at the Centre of Diagnosis and Treatment of Infertility in the Division of Infertility and Reproductive Endocrinology at the Poznan University of Medical Sciences. Follicular fluid was obtained by transvaginal ultrasound-guided aspiration, following the controlled ovarian stimulation according to designed protocol, as previously described [28].

After removal of the cumulus oophorus-oocytes-complexes, the freshly collected follicular fluid was centrifuged for 10 min at 250 g at room temperature (rt), and the density gradient centrifugation was performed. Briefly, the pellet of hGCs was suspended in Phosphate Buffered Saline (PBS) and centrifuged on 7 ml of Pancoll human (PAN-Biotech GmbH, Germany) for 20 min at 400 g. Separated GCs were aspirated by pipetting from the interphase layer, washed in 10 ml of Dulbecco’s Modified Eagle Medium (DMEM), centrifuged at 250 g for 10 min, and resuspended in cell culture medium [28].

After isolation, cells were seeded in cell culture flasks and initially maintained for 24 h in DMEM enriched with 10% Fetal Bovine Serum (FBS), supplemented with 10 mg/ml gentamicin, 10,000 μg/mL streptomycin, 10,000 U /ml penicillin, and 4 mM L-glutamine [28]. GCs were cultured in a humidified atmosphere of 5% CO_2_ and 95% air at 37 °C. After 24 h, cells were detached using a trypsin-EDTA solution and seeded in 96-wells U-bottom plates at a density of 10 000 viable cells/well. To enhance spheroids formation, cells were transferred to cell culture medium with methylcellulose (final concentration 0,25%) [29], centrifuged at 280 g for 10 min, and left for 72 h incubation. To ensure consistently sized hGCs spheroids, each of them was measured. The average diameter of the spheroids used in the experiments was 384.22 ± 24.15 μM.

### Cell viability assay

To evaluate the effect of the liposomal formulation of DMU-212 (lipDMU-212) in the spheroid model of GCs, the CellTiter-Glo® 3D Cell Viability Assay was performed. After 72 h of incubation of the formed spheroids, the tested compounds (DMU-212 and lipDMU-212) were added in the concentration range of 0-10 μM. DMU-212 was added from the stock solution prepared in DMSO. The final concentration of DMSO in cell treatment solutions was less than 0.1%. The liposomes solutions were added in the volumes, corresponding with the dilution factor as in the lipDMU-212 treatment. The spheroids were incubated with the compounds tested for 72 h. Then the assay was performed according to the manufacturer’s protocol. Briefly, equal volume of reagent was added into treated and untreated hGCs after 72 h, and incubated for 30 min. After that, the lysates were transferred to white 96-well plates for luminescence reading (Tecan Infinite 200 Pro).

### Steroidogenesis measurement

The secretion of estradiol and progesterone was determined using enzyme-linked immunosorbent assays, according to the manufacturer’s protocols. Briefly, after 24 h of preincubation period, the spheroids were maintained in cell culture medium containing 2% FBS and methylcellulose at a concentration of 0.25%, 10^−6^ M androstenedione as a substrate in the presence or absence of lipDMU-212 (0.039, 0.625 or 5 μM) and FSH (10 ng/ml and 100 ng/ml) for 72 h. The basal secretion of estradiol and progesterone was also analyzed; hGCs spheroids were cultured as described above with the exception of the addition of androstenedione and FSH. After the 72 h incubation time, supernatants were collected and stored at −20 °C until assayed. Quantification of the hormones was assessed by measuring the optical absorbance at 450 nm using an ELx - 800 microplate reader (BioTek Instruments, Winooski, VT, USA) and KC Junior software (BioTek Instruments). The lower detection limits and the intra-, and inter-assay coefficients of variation for estradiol were 10.6 pg/ml, 8.7–9.2 and 6.9–14.9%, while those of progesterone were 0.14 ng/ml, 5.4–7.0% and 4.3–10.0%, respectively. To meet the assay dynamic range, the samples were diluted 1:100 and 1:600 for estradiol and progesterone measurements, respectively. Subsequently, the dilution factor was taken into account for the calculation of the concentrations. The results obtained from the experiment were expressed in pg/ml for estradiol and ng/ml for progesterone.

### RNA isolation and RT-qPCR analysis

Samples from three independent experiments with three replicates per condition, collected after 72 h of treatment, were used for transcriptomic analysis using real-time quantitative polymerase chain reaction (RT-qPCR). Briefly, total RNA was extracted from human ovarian GCs using Tri Reagent as described elsewhere [30]. RNA concentration was quantified using a NanoDrop ND-1000 spectrophotometer (Nyxor Biotech, Paris, France). Subsequently, the total RNA was reversely transcribed into cDNA with the RT2 Easy First Strand Kit (Qiagen, Hilden, Germany) following the manufacturer’s instructions. RT-qPCR reactions were conducted in LightCycler® Instrument 96 (Roche Diagnostic, Mannheim, Germany) using SYBR® Green I (Master Mix Qiagen GmbH, Hilden, Germany), as a detection dye, and LightCycler Software 1.5 (Roche Diagnostic, Mannheim, Germany). The quantity of analyzed cDNA in each sample was standardized by housekeeping genes: *GAPDH* (glyceraldehyde-3-phosphate dehydrogenase), *HPRT* (hypoxanthine phosphoribosyltransferase) and *ACTB* (β-actin). Reaction was performed according to the thermocycling protocol: preincubation at 37 °C (30 s); 3 step amplification (95 °C—15 s, 59 °C—15 s, 72 °C—15 s) for 45 cycles; melting (95 °C—60 s, 40 °C—60 s, 70 °C—1 s, 95 °C—1 s); cooling at 37 °C (30 s). Target cDNA was quantified using the relative quantification method. For gene expression analysis, the 2 ∆∆Cq method was used [31]. Oligonucleotide sequences of primers used for RT-qPCR analyses are presented in Table 1.

**Table 1:** Oligonucleotide sequences of primers used for RT-qPCR analyses

Gene	Name	Primer sequence	Product Size (bp)
*CYP17A1*	Cytochrome P450 Family 17 Subfamily A Member 1	F: 5’-CAAGGATGGCGATCAGAAGC-3’ R: 5’-ACATTCAACTCAGGGTCCCC-3’	186
*CYP11A1*	Cytochrome P450 Family 11 Subfamily A Member 1	F: 5’-CTGTTGGATGCAGTGTCTCG-3’ R: 5’-TGGCATCAATGAATCGCTGG-3’	202
*HSD3B1*	Hydroxy-Delta-5-Steroid Dehydrogenase, 3 Beta- And Steroid Delta-Isomerase 1	F: 5’-GAAAGGTACCCAGCTCCTGT-3’ R: 5’-TACAGCCTTCTCAGCAAGCT-3’	193
*StAR*	Steroidogenic Acute Regulatory Protein	F: 5’-TCTCTACAGTGACCAGGAGC-3’ R: 5’-GAACACCTTGCCCACATCTG-3’	163
*CYP19A1*	Cytochrome P450 Family 19 Subfamily A Member 1; aromatase	F: 5’-ACATCTGGACAGGTTGGAGG-3’ R: 5’-TTGATGAGGAGAGCTTGCCA-3’	180
*HSD17B1*	Hydroxysteroid 17-Beta Dehydrogenase 1	F: 5’-CCACGTTGAGGGACCTGAAA-3’ R: 5’-CATTCACGTCCAGCACAGAG-3’	243
*ACTB*	Actin Beta	F: 5’-CCCTGGAGAAGAGCTACGAG-3’ F: 5’-CCCTGGAGAAGAGCTACGAG-3’	180
*HPRT*	Hypoxanthine Phosphoribosyltransferase 1	F: 5’-CCTGGCGTCGTGATTAGTGA-3’ R: 5’-GCCTCCCATCTCCTTCATCA-3’	162
*GAPDH*	Glyceraldehyde-3-Phosphate Dehydrogenase	F: 5’-CCAGAACATCATCCCTGCCT-3’ R: 5’-CCTGCTTCACCACCTTCTTG-3’	185

### Sodium dodecyl sulfate-polyacrylamide gel electrophoresis (SDS-PAGE) and western blotting analysis

After treatment, the hGCs spheroids were washed twice with PBS and the protein was extracted, using mix of RIPA lysis buffer and protease inhibitor, according to the manufacturer’s protocol. Subsequently, 10 μg of protein lysates were subjected to western blotting, resuspended in sample buffer, and separated using 4 to 20 % gradient Mini-PROTEAN® TGX™ gel by SDS-PAGE (120 V, 90 min). The protein was then transferred using the Trans-Blot Turbo Mini 0.2 µm PVDF Transfer Pack set, according to the manufacturer’s instructions. Blots were then incubated with primary rabbit antibodies: CYP11A1 (#14217, Cell Signaling Technology, Danvers, MA, USA) and HSD3B1 (#PA5-119789, Invitrogen, Waltham, MA, USA) in Tris-Buffered Saline Tween 0,1% (TBST) with 5% fat-free dry milk powder overnight, at 4 °C. Following several washes in TBST, immunodetection was performed using anti-rabbit secondary IgG, HRP-conjugated goat antibody (#7074, Cell Signaling Technology, Danvers, MA, USA), utilizing the iBright CL750 Imaging System and iBright Analysis Software (Invitrogen, Waltham, MA, USA). The PVDF membranes were incubated with anti-β-Actin (13E5) rabbit antibody (HRP conjugated) (#5125, Cell Signaling Technology, Danvers, MA, USA). Finally, Super Signal West Pico PLUS Chemiluminescent was used to reveal the bands. The obtained blots were further analyzed using the ImageJ software (U. S. National Institutes of Health, Bethesda, Maryland, USA).

### Statistical analysis

Statistical analysis was conducted using GraphPad Prism software v5.01. Data were collected in triplicate from at least three independent experiments. The results were shown as the means ± standard deviation (SD). Statistical studies for steroid hormones measurements were carried out using one-way analysis of variance (ANOVA) and the Student’s *t*-test post-hoc, while for the statistical analysis of RT-qPCR results Kruskal-Wallis and Tukey’s tests were used. Western blot bands were quantified using ImageJ software (U. S. National Institutes of Health, Bethesda, Maryland, USA). A *p*-value < 0.05 was considered statistically significant, although other *p*-values were also reported.

## Results

### Characterization of liposomal DMU-212 (lipDMU-212)

**Fig. 1:**
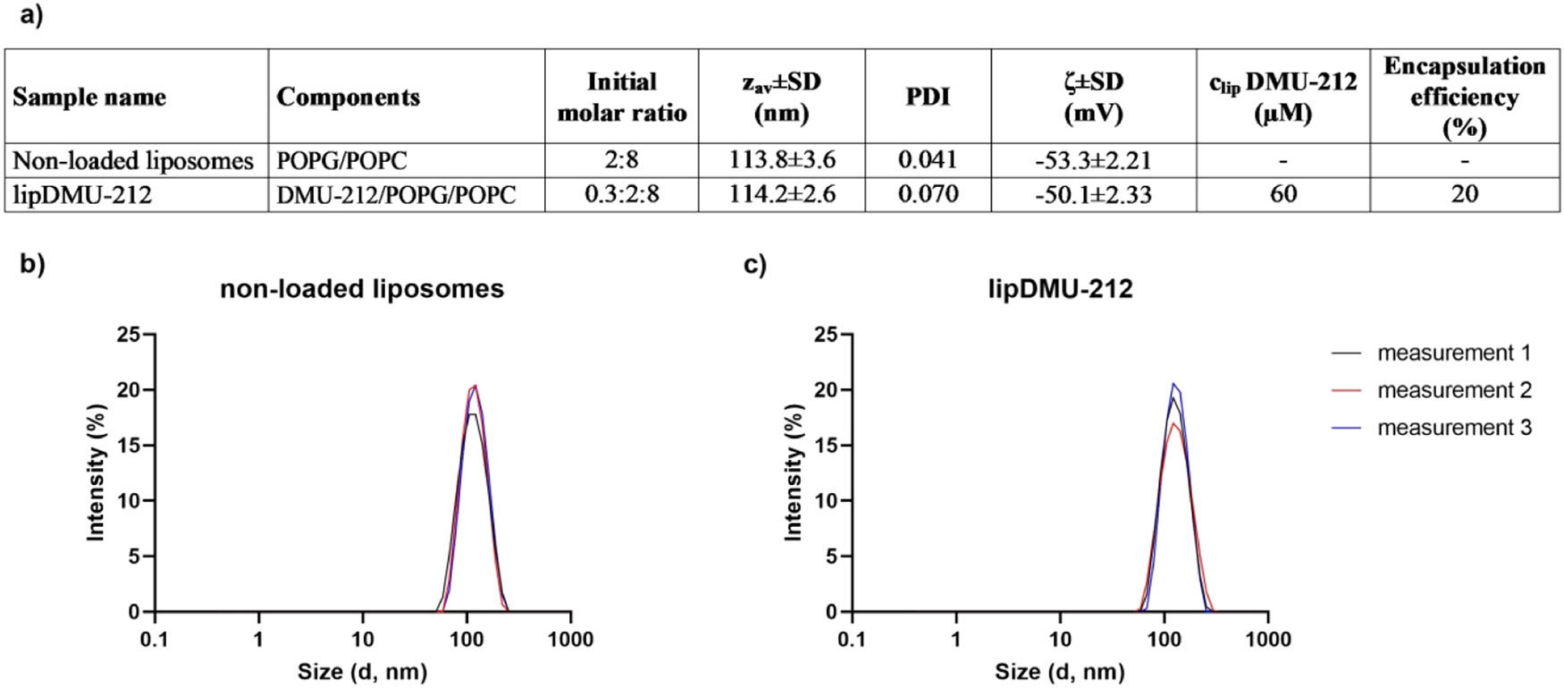
**a** Characteristics of non-loaded and lipDMU-212 liposomes used in the study: components, initial molar ratio, z-average (zav), polydispersity index (PDI), zeta potential (ζ), concentration of DMU-212 in liposomes (lipDMU-212) and encapsulation efficiency. Size and size distribution (data from three independent measurements) determined for **b**) non-loaded liposomes and **c**) lipDMU-212 liposomes

The characteristics of lipDMU-212 and non-loaded liposomes is presented in Fig. 1. The size of lipDMU-212 expressed as z-average was 114.2 ± 2.6 nm and was not significantly different than zav calculated for non-loaded liposomes (113.8 ± 3.6 nm). Both samples were characterized by homogeneous size distribution with a PDI below 0.1. The presence of POPG contributed to negative charge of the liposomal membrane with zeta potential −53.3 and −50.1 mV for non-loaded liposomes and lipDMU212, respectively. The concentration of DMU-212 in lipDMU-212 determined by HPLC was 60 µm and corresponded to *ca*. 20% encapsulation efficacy. For further studies lipDMU-212 liposomes were diluted in cell culture medium.

### Cell viability assay

**Fig. 2:**
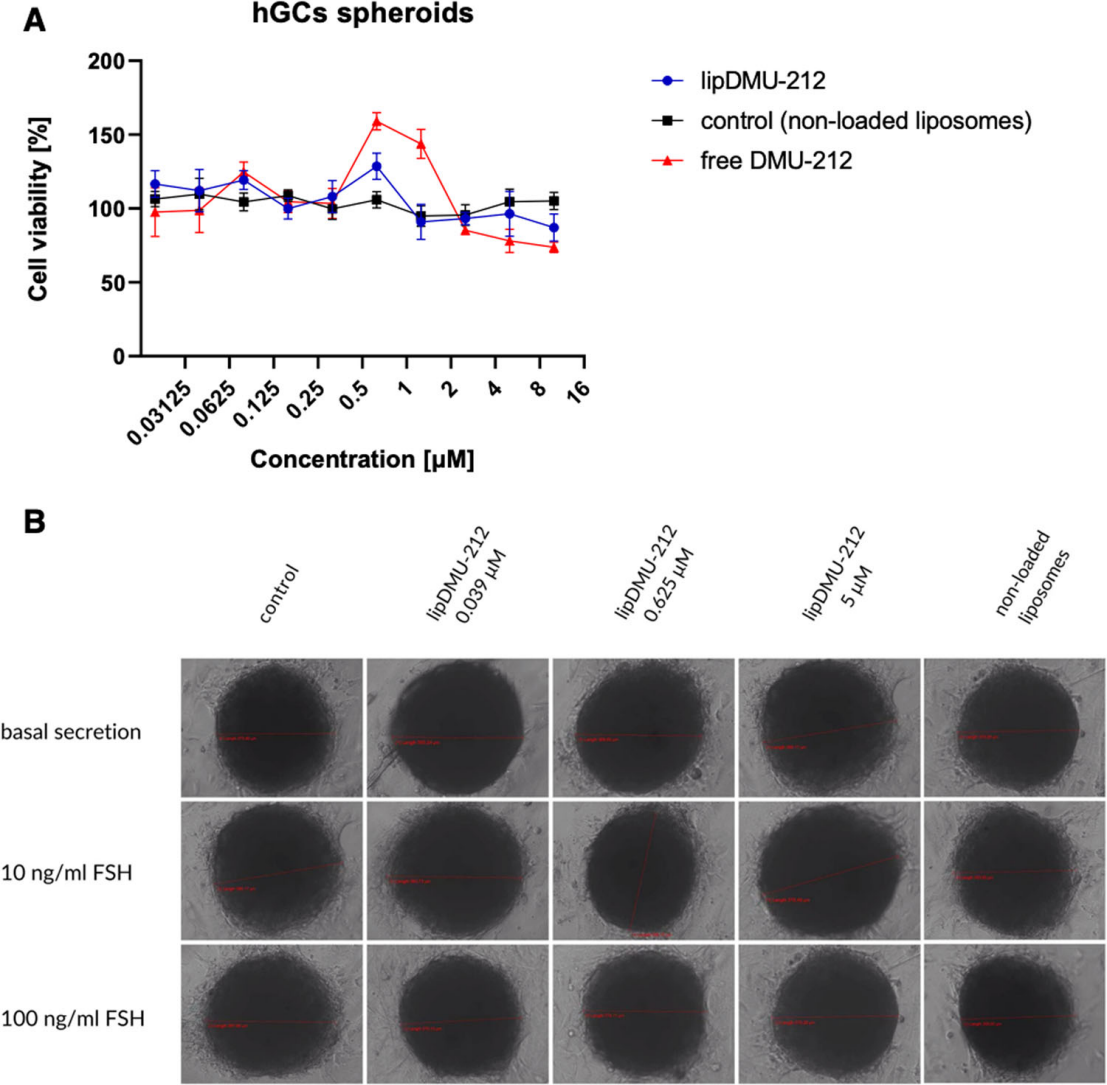
**A** The effect of free DMU-212 and lipDMU-212 on human ovarian GCs spheroids’ viability. As a control, the activity of non-loaded liposomes was evaluated. The liposomes solution was diluted by the same dilution factor as the liposomal formulation of DMU-212. The cell viability was assessed following 72 h incubation with tested compound and its liposomal formulation. Results of three independent replicates are presented as mean ± SD. **B** Spheroids morphology upon treatment with lipDMU-212 (at the concentrations of 0.039 μM, 0.625 μM, and 5 μM) with or without FSH (10 or 100 ng/ml) and 10^−6^ M androstenedione as a substrate. The images were acquired after 72 h of treatment

Cell viability studies showed no cytotoxicity of the liposomal formulation of lipDMU-212 in the concentration range of 0.0–10.0 μM on human ovarian GCs spheroids after 72 h of treatment. No pro- and anti-proliferating activities of non-loaded liposomes in hGCs spheroids were observed. Interestingly, a statistically significant increase in GCs proliferation after 72 h incubation with lipDMU-212 and DMU-212 at the lower concentrations was noted. As shown in Fig. 2A, lipDMU-212 caused an up-regulation in cell viability by ~25, ~20, and ~15%, at the concentrations of 0.6250 μM, 0.0781 μM and 0.0195 μM, respectively. Correspondingly, DMU-212 was also revealed to increase the proliferation of hGCs; the most pronounced effect (~60 %) was shown after treatment with 0.625 μM of DMU-212. Nevertheless, after incubation with 1.25 μM and 0.078 μM of DMU-212, the cell viability increased by ~40 and ~25%, respectively. On the contrary, decreased viability of hGCs by ~15–25% was observed at the concentration range of 2.5–10 μM of DMU-212. The spheroids’ morphology is presented in Fig. 2B.

### Effect of lipDMU-212 on the basal secretion of estradiol and progesterone in human luteinized GCs spheroids

**Fig. 3:**
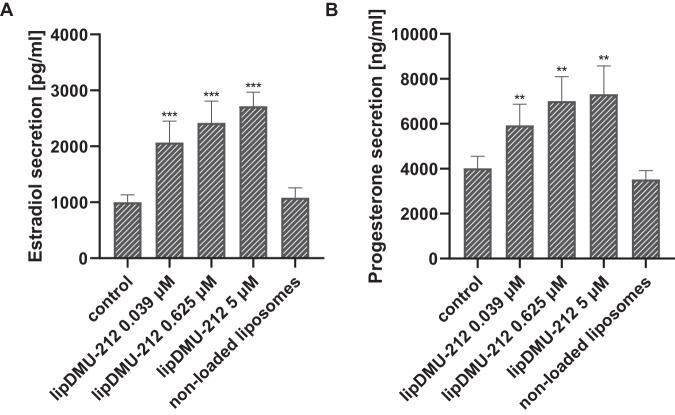
The effect of lipDMU-212 on basal secretion of estradiol (**A**) and progesterone (**B**) from human luteinized GCs’ spheroids after 72 h of culture. Results of three independent replicates are presented as mean ± SD. ***p < 0.001, **p < 0.01, *p < 0.05 indicate a significant difference from the control

The estradiol and progesterone levels were measured in spent culture media after 72 h of treatments with lipDMU-212 at three different concentrations (0.039 μM, 0.625 μM, and 5 μM). As shown in Fig. 3, lipDMU-212 significantly enhanced hormone secretion in a dose-dependent manner (*p* < 0.001 and *p* < 0.01), as compared to the controls for all doses tested for estradiol and progesterone assays, respectively. The most pronounced effects were observed after treatments with the highest concentration of lipDMU-212; the estradiol secretion increased to 2719,45 ± 247,94 pg/ml, compared to the control (1000,13 ± 136,04 pg/ml) while the progesterone level increased to 7319,41 ± 1263,04 ng/ml vs. 4024,21 ± 533,028 ng/ml for the control. There were no significant differences after treatment with non-loaded liposomes (*p* = 0,3980 for estradiol and *p* = 0,1617 for progesterone assessment), as compared to the control.

### Effect of lipDMU-212 on 10 ng/ml FSH-stimulated secretion of estradiol and progesterone in human luteinized GCs spheroids

**Fig. 4:**
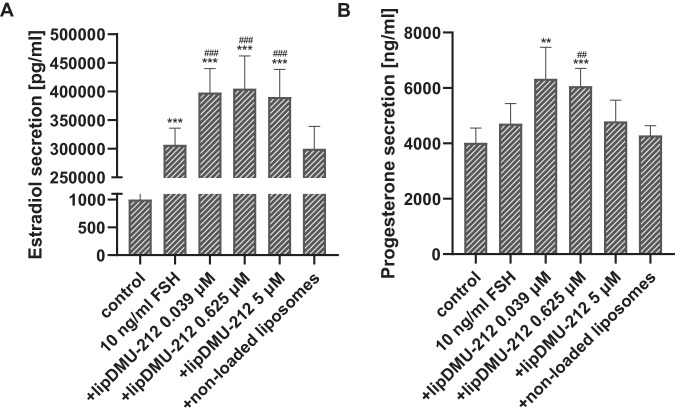
The effect of lipDMU-212 on 10 ng/ml FSH-stimulated secretion of estradiol (**A**) and progesterone (**B**) in human luteinized GCs’ spheroids after 72 h of culture. Results of three independent replicates are presented as mean ± SD. ***p < 0.001, **p < 0.01, *p < 0.05 indicate a significant difference from the control, and ^###^*p* < 0.001, ^##^*p* < 0.01, ^#^*p* < 0.05 indicate a significant difference from the control treated with 10 ng/ml FSH

As presented in Fig. 4, stimulation of the hGCs spheroids with 10 ng/ml FSH was revealed to increase estradiol secretion ~3000 times (*p* < 0.001), while progesterone secretion was not affected (*p* = 0.1420). lipDMU-212 caused a significant up-regulation in estradiol levels by ~30% (*p* < 0.001) after treatments with all three concentrations tested combined with FSH stimulation. Regarding progesterone level, only one treatment, lipDMU-212 at the concentration of 0.625 μM, significantly enhanced progesterone secretion (*p* < 0.01; 6072,69 ± 629,69 ng/ml vs. 4218,31 ± 684,89 ng/ml for control). No statistically significant differences after treatment with non-loaded liposomes, compared to controls (*p* = 0.7160 for estradiol and *p* = 0.2877 for progesterone evaluation), were shown.

### Effect of lipDMU-212 on 100 ng/ml FSH-stimulated estradiol and progesterone secretion in human luteinized GCs spheroids

**Fig. 5:**
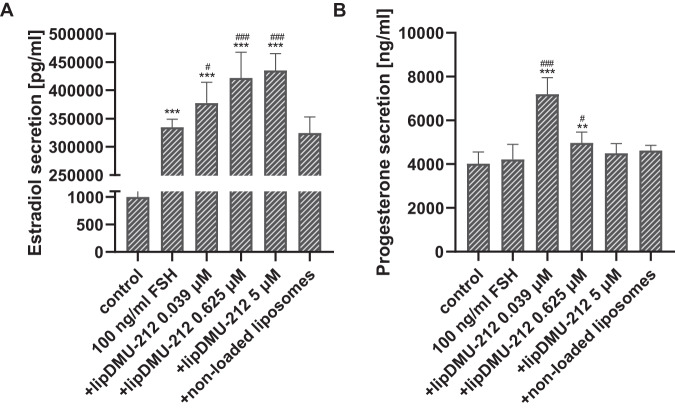
The effect of lipDMU-212 on 100 ng/ml FSH-stimulated secretion of estradiol (**A**) and progesterone (**B**) from human luteinized GCs’ spheroids after 72 h of culture. Results of three independent replicates are presented as mean ± SD. ***p < 0.001, **p < 0.01, *p < 0.05 indicate a significant difference from the control, and ^###^*p* < 0.001, ^##^*p* < 0.01, ^#^*p* < 0.05 indicate a significant difference from the control treated with 100 ng/ml FSH

After 72 h of treatment, stimulation with FSH at the concentration of 100 ng/ml significantly increased the estradiol secretion (334576,89 ± 14242,13 pg/ml vs. 1000,13 ± 136,04 for control) (Fig. 5). Concomitantly, the secretion of progesterone remained unchanged. lipDMU-212 when combined with FSH was shown to enhance estradiol secretion at all the concentrations in a dose-dependent manner and the most pronounced effect was noted after treatment with lipDMU-212 at the concentration of 5 μM (435096,27 ± 29807,77 pg/ml). An increase in progesterone levels after treatment of 100 ng/ml FSH-stimulated hGCs spheroids with lipDMU-212 at concentrations of 0.039 and 0.625 μM (7195,07 ± 755,98 ng/ml, *p* < 0.001; 4973,79 ± 485,27 ng/ml, *p* < 0.05, respectively) was observed. There were no statistically significant changes after treatment with non-loaded liposomes, compared to controls (*p* = 0.3908 for estradiol and *p* = 0.1646 for progesterone assessment).

### Effect of lipDMU-212 on the expression of genes involved in steroidogenesis

**Fig. 6:**
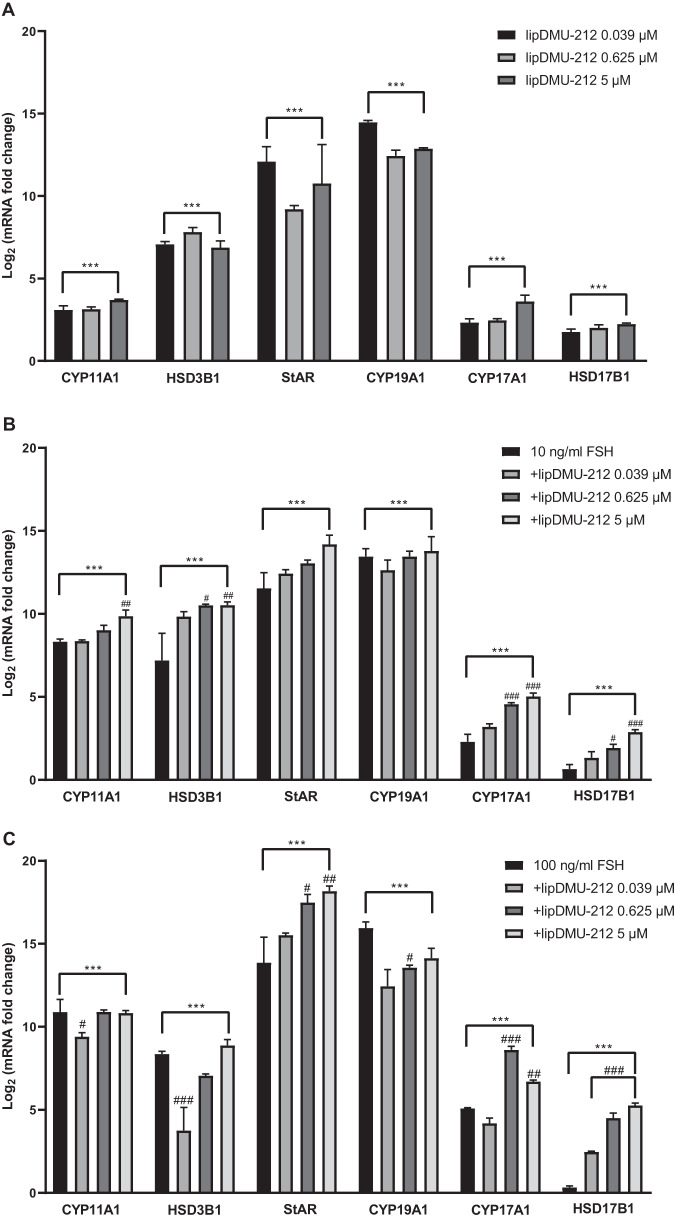
The expression of steroidogenesis-related genes after 72 h of treatment with (**A**) lipDMU-212 (0.039 μM, 0.625 μM, and 5 μM), (**B**) 10 ng/ml FSH and lipDMU-212 (0.039 μM, 0.625 μM, and 5 μM), and (**C**) 100 ng/ml FSH and lipDMU-212 (0.039 μM, 0.625 μM, and 5 μM). Results of three independent replicates are presented as mean ± SD. ***p < 0.001, **p < 0.01, *p < 0.05 indicate a significant difference from the control, ^###^*p* < 0.001, ^##^*p* < 0.01, ^#^*p* < 0.05 indicate a significant difference from the control treated with 10 ng/ml FSH (**B**) and 100 ng/ml FSH (**C**)

To clarify the signaling pathway by which lipDMU-212 enhances steroidogenesis in hGCs, the expression of several genes involved in estradiol and progesterone secretion was studied (Fig. 6). Increased expression of 6 steroidogenesis-related genes in hGCs exposed to lipDMU-212 were observed, in all concentrations tested compared to untreated control. The most pronounced up-regulation was noted in the level of *CYP19A1* transcript whose expression was increased by ~14.5, ~12.5, and ~13 times after treatment with lipDMU-212 in concentrations of 0.039 μM, 0.625 μM, and 5 μM, respectively. lipDMU-212 in concentrations 0.625 μM and 5 μM induced the increase in *StAR* expression 10-fold, while the expression after exposure to the lowest one was increased ~12 times. The expression of *HSD3B1* was upregulated ~7 times after treatment with all concentrations of lipDMU-212. The transcript levels of *CYP11A1*, *CYP17A1*, and *HSD17B1* were slightly elevated after treatment with all concentrations of lipDMU-212 (~3–4, ~2–3, and ~2 times, respectively).

The addition of 10 ng/ml FSH and 10^−6^ M androstenedione to the culture medium significantly upregulated the transcript levels of all 6 tested genes when compared to the control (Fig. 6B). Furthermore, lipDMU-212 was found to act synergistically and improve FSH activity by increasing *CYP17A1* and *HSD17B*1 mRNA transcript levels in a dose-dependent manner. The combination of lipDMU-212 (0.625 μM and 5 μM) and FSH increased the transcript levels of *CYP11A1* ~ 9 and ~10 times compared to untreated control, while the addition of FSH was noted to increase the expression 8-fold. Similarly, lipDMU-212 in all concentration tested upregulated the expression of *HSD3B1* ~ 10 times when the expression after treatment with FSH alone was increased 7-fold. CYP19A1 expression did not increase significantly after additional lipDMU-212 treatment compared to the 10 ng/ml FSH control.

Similarly, in cells stimulated with 100 ng/ml FSH, the expression of the tested genes was significantly elevated compared to the untreated control (Fig. 6C). lipDMU-212 was found to enhance FSH activity by increasing *StAR* and *HSD17B1* expression in a dose-dependent manner. CYP11A1 was down-regulated after treatment with the combination of 100 ng/ml FSH and lipDMU-212 at the concentrations of 0.039 μM, compared to the 100 ng/ml FSH control. There was no significant difference after combined treatment with 100 ng/ml FSH and lipDMU-212 at the concentrations of 0.625 μM and 5 μM. Interestingly, lower concentrations of lipDMU-212 when combined with 100 ng/ml FSH, decreased expression level of *HSD3B1*. Similarly, lipDMU-212 combined with FSH down-regulated the expression of *CYP19A1* compared to the control treated with FSH alone. lipDMU-212 was found to enhance FSH activity and increase *CYP17A1* expression, but only at concentrations of 0.625 μM and 5 μM.

### Protein expression analysis

**Fig. 7:**
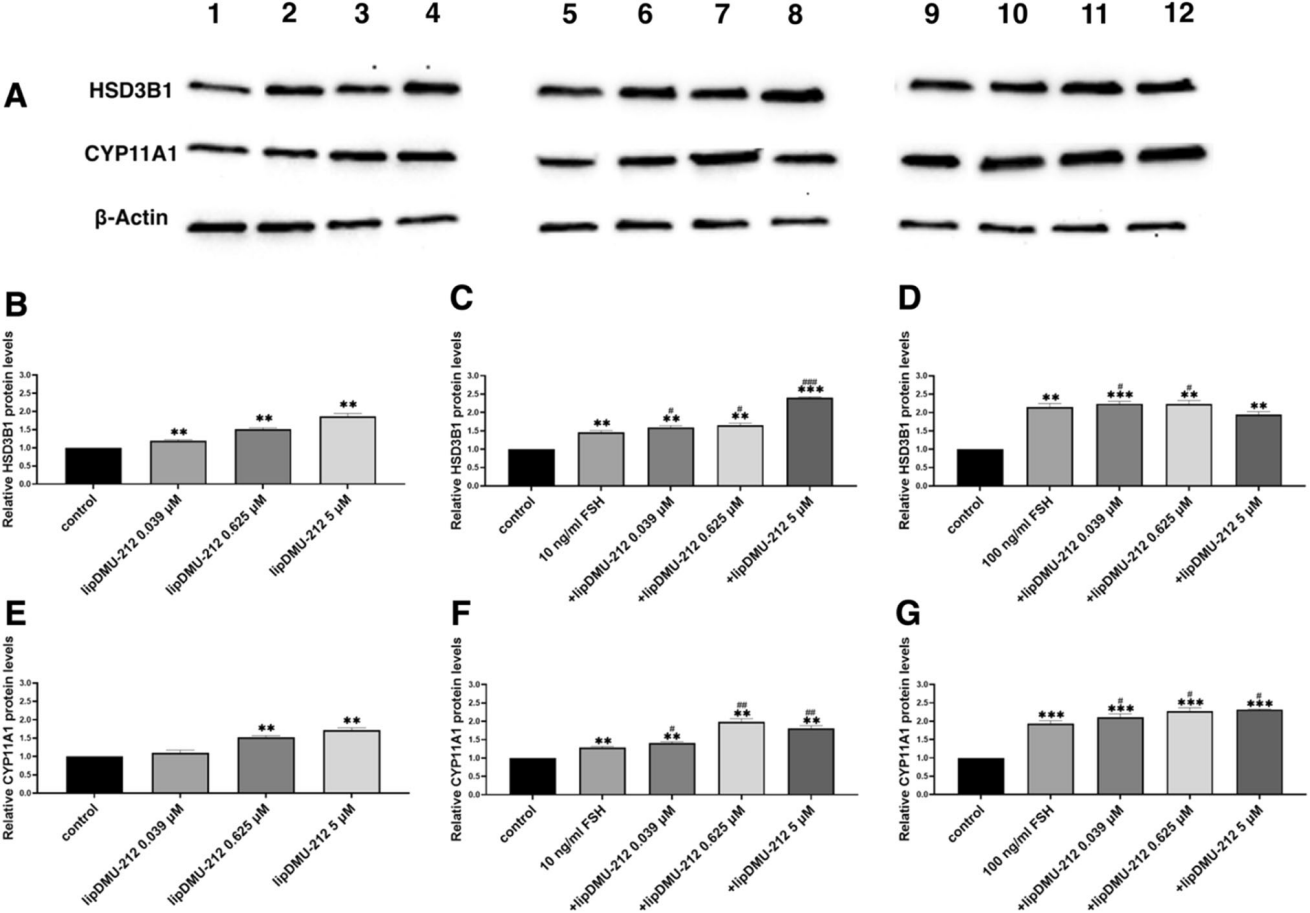
**A** Western blot analysis of HSD3B1 and CYP11A1 protein levels in hGCs treated for 72 h with (1) vehicle, (2) 0.039 μM lipDMU-212, (3) 0.625 μM lipDMU-212, (4) 5 μM lipDMU-212, (5) 10 ng/ml FSH, (6) 10 ng/ml FSH and 0.039 μM lipDMU-212, (7) 10 ng/ml FSH and 0.625 μM lipDMU-212, (8) 10 ng/ml FSH and 5 μM lipDMU-212, (9) 100 ng/ml FSH, (10) 100 ng/ml FSH and 0.039 μM lipDMU-212, (11) 100 ng/ml FSH and 0.625 μM lipDMU-212, (12) 100 ng/ml FSH and 5 μM lipDMU-212. **B**, **E** The effect of 72 h treatment with lipDMU-212 (0.039 μM, 0.625 μM, and 5 μM) on HSD3B1 and CYP11A1 protein expression, respectively. **C**, **F** The effect of 72 h co-treatment with lipDMU-212 (0.039 μM, 0.625 μM, and 5 μM) and 10 ng/ml FSH on expression of the proteins HSD3B1 and CYP11A1, respectively. **D**, **G** The effect of 72 h co-treatment with lipDMU-212 (0.039 μM, 0.625 μM, and 5 μM) and 100 ng/ml FSH on expression of the proteins HSD3B1 and CYP11A1, respectively. Results of three independent replicates are presented as mean ± SD. ***p < 0.001, **p < 0.01, *p < 0.05 indicate a significant difference from the control. ^###^*p* < 0.001, ^##^*p* < 0.01, ^#^*p* < 0.05 indicate a significant difference from the control treated with 10 ng/ml FSH (**C**, **F**) and from the control treated with 100 ng/ml FSH (**D**, **G**)

Western blot was employed to analyze the effect of lipDMU-212 and FSH treatments on levels of CYP11A1 and HSD3B1 proteins (Fig. 7). Our study showed that treatment with lipDMU-212 alone increased the expression of HSD3B1 and CYP11A1 proteins in a dose-dependent manner, compared to the untreated control. Treatments with 10 ng/ml FSH as well as the combination of 10 ng/ml FSH and lipDMU-212 at the lower concentrations tested caused an up-regulation in the level of HSD3B1 by ~50%. The most pronounced effects (~95–140% increase) were observed after treatments with combination of 10 ng/ml FSH and higher concentration of the test compound, 100 ng/ml FSH alone and combined with lipDMU-212 at all concentrations tested.

In cells treated with 10 ng/ml FSH alone and 10 ng/ml FSH combined with 0.039 μM lipDMU-212, we found an increase in CYP11A1 protein levels by ~30 and ~40%, respectively. Moreover, co-treatment with the highest concentration of lipDMU-212 and 10 ng/ml FSH as well as the treatments with 100 ng/ml FSH alone and combined with lipDMU-212 in all concentrations tested, enhanced the protein expression by ~100%.

## Discussion

Due to the fact that DMU-212 is insoluble in water, in the study, DMU-212 was applied in the liposomal formulation. The carrier-mediated administration increases DMU-212 concentration in the water environment and prevents precipitation.

2D cell culture models are assumed not to reflect in vivo situation entirely. Concomitantly, 3D is referred to as a bridge between flat in vitro and in vivo experimental parts. Among a plethora of benefits that the 3D culture holds are: appropriate cell polarity, more realistic distribution of nutrients and oxygen, and gene and protein expression levels [32]. Hence, in the present study, we assessed the steroidogenic activity of DMU-212 in 3D hGCs in vitro model.

In the first stage of our study, we performed a cell viability assay, which showed no cytotoxicity of the liposomal formulation of DMU-212 at the concentration range of 0.0–10.0 μM on hGCs spheroids after 72 h of treatment. Interestingly, a significant increase in cell viability after treatment with both free DMU-212 and lipDMU-212 was noticed. The most pronounced effect was revealed in cells treated with free DMU-212 and lipDMU-212 at the concentration of 0.625 µm, which might suggest their prominent proliferative effect on hGCs. Hence, 0.625 µm of lipDMU-212 was selected for further studies. Moreover, based on the obtained results, we chose two non-toxic concentrations (0.039 and 5 µM) of the compound tested.

GCs play numerous significant roles in ovaries, including the secretion of steroid hormones. In our research, we measured the estradiol and progesterone levels in hGCs treated with lipDMU-212. These analyses revealed that lipDMU-212 significantly enhanced both estradiol and progesterone secretion in a dose-dependent manner. To the best of our knowledge, there is no available data in the literature regarding how DMU-212 influences steroid hormones secretion. However, the activity of several hydroxylated and methylated resveratrol analogs on swine GCs has been studied [25]. In the mentioned study, hydroxylated resveratrol analog (2-hydroxy-3,5,4’-trimethoxystilbene) was shown to stimulate both estradiol and progesterone secretion while methylated one enhanced production of only estradiol. Contrary to our results, Basini et al. have noted decreased progesterone levels at the highest concentration tested (100 μM) [25]. Nevertheless, the results of our study are consistent with those of Moreira-Pinto et al., which showed an increase in estradiol secretion after 72 h of treatment with 5 μM resveratrol [33].

It is widely known that ERα and ERβ receptors mediate the effects of estrogens and play pivotal roles in various physiological and pathological processes [34]. According to previous research on the steroidogenic activity of resveratrol, it has a similar affinity to estrogen receptors ERα or ERβ and has the ability to interfere with estradiol functions [35]. Despite various conflicting results, resveratrol is known as an estrogen mixed agonist/antagonist [35, 36]. Recently, Kobylka et al. have studied the ability of several methoxy- and hydroxy- resveratrol analogs to bind to the estrogen receptors [37]. Interestingly, the study revealed that DMU-212 does not exert binding affinity to ERα and Erβ [37]. The possible explanation of these results may be the lack of hydroxy groups in the structure of DMU-212 as -OH groups are suggested to possibly be responsible for binding estrogen receptors [38]. In the present study, we showed enhanced steroidogenesis after lipDMU-212 treatment. Hence, the compound tested might be suggested to influence the secretion of steroid hormones through a different mechanism than binding to ER receptors. The expression of several CYP450 genes, e.g., CYP1A1 and CYP1B1, is under the control of aryl hydrocarbon receptor (AhR) [39]. AhR is a ligand-activated member of the basic helix-loop-helix (bHLH)-PAS transcription factors, which participates in various physiological and pathological processes, also within the reproductive organs [40, 41]. In women with PCOS, a correlation between elevated levels of *AhR*, *ARNT*, *CYP1A1*, and *CYP1B1* genes and clinical hyperandrogenism, follicular fluid testosterone levels, and disturbed folliculogenesis was observed [41]. Since high levels of *AhR, CYP1A1*, and *CYP1B1* genes are correlated with impaired folliculogenesis, the inhibition of *AhR* signaling pathway might be considered as a novel treatment approach for PCOS patients [42]. Interestingly, our previous study showed that DMU-212 suppressed the AhR protein expression in the nuclear fraction of ovarian cancer A-2780 cells and their non-cancerous counterparts [24]. Hence, we suggest that DMU-212 loaded into liposomes may be potentially used in the treatment of ovarian dysfunctions.

To determine the signaling pathways related to the steroidogenic activity of lipDMU-212, we performed RT-qPCR analyses targeting selected genes involved in estradiol and progesterone production.

We noticed that lipDMU-212 remarkably evoked the up-regulation of the expression of *StAR*. StAR is known to control cholesterol transfer within the mitochondria, which is the rate-limiting process in the secretion of steroid hormones [43]. It is common knowledge that granulosa and theca cells work together in the biosynthesis of ovarian hormones. This process is described by the “two-cell, two-gonadotropin theory”, which holds that ovarian hormones are produced from cholesterol through intricate interactions between the granulosa and theca cells [1]. At this point, cholesterol is converted into pregnenolone by CYP11A1. Then pregnenolone is transferred from mitochondria to the smooth endoplasmic reticulum, where it could be transformed into progesterone and dehydroepiandrosterone (DHEA) by 3β-HSD and 17β -hydroxylase/17,20-desmolase (CYP17A1), respectively. The latter ones catalyze the conversion of DHEA and progesterone to androstenedione. In the next step, androstenedione might be converted to testosterone in theca cells by 17β -hydroxysteroid dehydrogenase (17β-HSD) or translocated into the GCs, where in turn CYP19A1 converts androstenedione and testosterone to estrone and estradiol, respectively. Moreover, 17β-HSD might also produce estradiol using estrone as a substrate [44–47].

In our study, we found a significant increase in the transcript levels of *CYP11A1, HSD3B1, CYP17A1, CYP19A1*, and *HSD17B1* in GCs treated with lipDMU-212. These genes are known to encode the entire steroidogenesis cascade in theca and granulosa cells. Hence, we suggest that the mechanism of steroidogenic actions of lipDMU-212 may be associated with their enhanced expression.

CYP19A1 is another enzyme that is crucial for estrogen synthesis as well as ovarian development. In GCs, aromatase catalyzes the demethylation of androstenedione and testosterone, leading to the A ring aromatization, which finally results in production of estrone and estradiol, respectively. Before menopause, estrogen is derived primarily from ovarian GCs and the placenta in healthy women [48]. Moreover, the expression of *CYP19A1* is then higher in GCs compared to other tissues [49]. The expression of aromatase is controlled in a tissue-specific manner *via* several distinct promoters, which alter its expression by recruiting different transcription factors [50]. The proximal promoter II (PII) has been revealed to play an important role in the regulation of aromatase expression in the ovary while the role of PI.1 is minor, however, its transcription is up-regulated during the luteinization of GCs [48, 51]. Previous research has shown that the transcription of the *CYP19A1* promoter in GCs might be altered by several factors. Solak et al. have revealed that many phytoestrogens enhance the expression of *CYP19A1* through the activation of ovarian-specific promoters I.3 and II [52]. Since DMU-212 is structurally similar to an estrogen scaffold, we suggest that the mechanism of lipDMU-212 steroidogenic activity might be also related to the activation of specific ovarian promoters.

In the present study, the most pronounced increase was found in *CYP19A1* and *StAR* transcript levels in GCs treated with lipDMU-212 in the lowest concentration tested (0.039 μM). Correspondingly, Morita et al. have demonstrated an increase in the expression of *StAR* and *CYP19A1* in rat GCs, however, after treatment with resveratrol at a higher concentration (100 μM) [53].

FSH is a glycoprotein hormone produced by the anterior pituitary in response to gonadotropin-releasing hormone (GnRH) from the hypothalamus [54]. One of the key actions of FSH is the induction of aromatase expression [54].

It has been revealed that the activity of FSH in GCs depends on the cyclic adenosine monophosphate (cAMP), and the regulation of aromatase activity is associated with the activation of phosphatidylinositol 3-kinase (PI3K) and extracellular signal-regulated kinase (ERK) signaling pathways [48]. In addition, several studies have reported that FSH stimulates estrogen production of GCs when the cells are provided with an aromatizable substrate [55]. In the present study, similar to the results of Moreira-Pinto et al. [33], the addition of 10 ng/ml or 100 ng/ml FSH and androstenedione resulted in a significant increase (over 3000-fold) in estradiol secretion compared to the untreated control.

Moreover, the combined treatment with 10 ng/ml FSH and lipDMU-212 in all tested concentrations caused an up-regulation in estradiol levels by ~30% compared to cells treated with 10 ng/ml FSH alone, and a dose-dependent increase in estradiol production after the treatments with a combination of lipDMU-212 and 100 ng/ml FSH. Hence, it can be assumed that lipDMU-212 works synergistically and enhances the activity of FSH. Regarding progesterone levels, a notable increase was found after combined treatment with 10 ng/ml FSH and lipDMU-212 at the concentration of 0.625 μM. The higher concentration of FSH (100 ng/ml) combined with respectively 0.039 μM and 0.625 μM lipDMU-212 has also been shown to upregulate progesterone levels significantly. Surprisingly, we found that the expression of genes involved in the progesterone synthesis, CYP11A1, and HSD3B1, was decreased after combined treatment with 0.039 μM lipDMU-212 and 100 ng/ml FSH as compared to the 100 ng/ml FSH control. To clarify this issue, we investigated the levels of CYP11A1 and HSD3B1 proteins since mRNA expression has been shown to not always reflect in protein level due to the complexity of the regulation of gene expression [56]. Contrary to the results of the mRNA analysis, we showed that co-treatment with 0.039 μM lipDMU-212 and 100 ng/ml FSH slightly (*p* < 0.05) increased protein levels of HSD3B1 and CYP11A1 as compared to the control treated only with 100 ng/ml FSH. In this regard, our findings are in line with increased progesterone secretion after combined treatment with 0.039 μM lipDMU-212 and 100 ng/ml FSH.

To summarize, we suggest that the mentioned increase in the steroid hormones’ secretion might be correlated with the up-regulation of *CYP11A1, HSD3B1, StAR, CYP17A1, and HSD17B1* expression after combined treatments with 0.625 μM as well as 5 μM lipDMU-212 and 10 ng/ml FSH. Furthermore, the elevated level of estradiol and progesterone of GCs treated with both 100 ng/ml FSH and higher concentrations of lipDMU-212 might be related to increased expression of *StAR, CYP17A*, and *HSD17B1*.

## Conclusions

Our study showed for the first time that liposomal formulation of DMU-212 enhanced both estradiol and progesterone secretion in human luteinized ovarian GCs. We reported a remarkable increase in transcript levels of steroidogenesis-related genes, especially *StAR* and *CYP19A1*, which encodes proteins crucial for estradiol secretion. Thus, our results suggest that lipDMU-212 might be potentially beneficial in the treatment of disorders related to estrogen deficiency and hyperandrogenism, such as PCOS.
